# Ocular coloboma—a comprehensive review for the clinician

**DOI:** 10.1038/s41433-021-01501-5

**Published:** 2021-03-21

**Authors:** Gopal Lingam, Alok C. Sen, Vijaya Lingam, Muna Bhende, Tapas Ranjan Padhi, Su Xinyi

**Affiliations:** 1 National University Hospital, Singapore, Singapore; 2 Department of Ophthalmology, Yong Loo Lin School of Medicine, National University of Singapore, Singapore, Singapore; 3 Singapore Eye Research Institute (SERI), Singapore, Singapore; 4 Sadguru Netra Chikitsalaya, Chitrakoot, India; 5 Medical Research Foundation, Chennai, India; 6 L V Prasad Eye Institute, Bhubaneswar, India; 7 Institute of Molecular and Cell Biology (IMCB), Agency for Science, Technology and Research (A*STAR), Singapore, Singapore

**Keywords:** Retinal diseases, Eye abnormalities

## Abstract

Typical ocular coloboma is caused by defective closure of the embryonal fissure. The occurrence of coloboma can be sporadic, hereditary (known or unknown gene defects) or associated with chromosomal abnormalities. Ocular colobomata are more often associated with systemic abnormalities when caused by chromosomal abnormalities. The ocular manifestations vary widely. At one extreme, the eye is hardly recognisable and non-functional—having been compressed by an orbital cyst, while at the other, one finds minimalistic involvement that hardly affects the structure and function of the eye. In the fundus, the variability involves the size of the coloboma (anteroposterior and transverse extent) and the involvement of the optic disc and fovea. The visual acuity is affected when coloboma involves disc and fovea, or is complicated by occurrence of retinal detachment, choroidal neovascular membrane, cataract, amblyopia due to uncorrected refractive errors, etc. While the basic birth anomaly cannot be corrected, most of the complications listed above are correctable to a great extent. Current day surgical management of coloboma-related retinal detachments has evolved to yield consistently good results. Cataract surgery in these eyes can pose a challenge due to a combination of microphthalmos and relatively hard lenses, resulting in increased risk of intra-operative complications. Prophylactic laser retinopexy to the border of choroidal coloboma appears to be an attractive option for reducing risk of coloboma-related retinal detachment. However, a majority of the eyes have the optic disc within the choroidal coloboma, thus making it difficult to safely administer a complete treatment.

## 摘要

典型的眼组织缺损是由胚胎裂闭合不全引起的。眼组织缺损可以为偶发、遗传性 (已知或未知的基因缺陷)或与染色体异常有关。眼组织缺损多与染色体异常引起的全身异常有关。眼部表现差异多样。比如被眼眶囊肿压迫时, 其中一眼几乎无法视物、视觉功能丧失, 而另一只眼的结构和功能却丝毫不受影响。眼底的变异性则涉及到组织缺损的大小(前后和横向的范围)以及视盘和黄斑中心凹的受累程度。当组织缺损累及视盘和黄斑的中心凹时, 或合并视网膜脱离、脉络膜新生血管膜、白内障、因屈光不正引起的弱视等, 则会影响视力。虽然眼组织先天性异常无法治疗, 但上述大多数并发症在很大程度上是可以治疗的。目前, 组织缺损相关性视网膜脱离的手术治疗已经取得了良好的效果。但由于先天性小眼球和相对坚硬的晶状体, 白内障手术具有一定的挑战性, 从而增加了术中并发症的风险。脉络膜缺损边缘的预防性激光光凝治疗对于降低组织缺损相关性视网膜脱离的发病风险是一种不错的选择。然而, 大部分患眼的视盘都在脉络膜的缺损范围内, 因此很难安全的进行完整的治疗。

## Introduction

Coloboma is a Greek word that means mutilation [1]. Coloboma of the fundus is caused by defective closure of the embryonal fissure. Typical coloboma is the term used to describe the defects seen in the inferior/infero-nasal part of the fundus that can be clearly attributed to defect in closure of embryonal fissure. Similar defects seen elsewhere have been termed atypical colobomata. Complete coloboma describes defects involving the optic disc, choroid/retina, ciliary body, zonules, lens (notching) and iris. In extreme cases, cysts can develop from the margin of the coloboma and extend into the orbit, making the eye functionless, while subtle involvement such as mild hypoplasia of iris, a notch in the pupillary border, etc., have no effect on the function of the eye. Fundus coloboma can be associated with variable degree of microphthalmos. Sporadic coloboma is common, although several systemic abnormalities and syndromes have been described, for some of which genetic loci have been identified.

Fundus coloboma poses threat to vision by way of the involvement of the macula and optic disc in the coloboma as well the increased risk of retinal detachment (RD) during the lifetime of the individual. The management of coloboma-related RDs has undergone significant changes with the advent of pars plana vitrectomy techniques and currently yields good reproducible results.

### Relevant embryology

The eye field that ultimately develops into the visual system is specified in the medial anterior neural plate very soon after gastrulation in 3rd week of gestation [2]. The optic vesicles form as outpouching of the forebrain. The surface ectoderm near the optic vesicle forms the lens placode and then into the lens vesicle. The distal part of the optic vesicle represents the future neural retina while the proximal part represents the future retinal pigment epithelium (RPE). The optic vesicle invaginates to form a double-layered optic cup. The distal part of the vesicle becomes the inner layer of the optic cup and develops into neural retina, while the outer layer derived from the proximal part of the optic vesicle forms the RPE. A ventral invagination along the optic cup and optic stalk termed the embryonal/choroidal/foetal fissure permits the mesenchyme to enter the optic cup. This fissure closes normally by 5–7 weeks of gestation (around 17-mm stage).

Several eye field transcription factors such as *PAX6, SIX3, LHX2, and RAX* in different combinations are needed at each stage of development of the eye [3]. Transcription factors intrinsic to cells interact with and modulate extrinsic signals. The optic vesicle contains retinal stem cells that are competent to become neuro-sensory retinal cells, RPE, or optic stalk depending on the appropriate combination of signals. At the stage of optic vesicle, the presumptive neural retina (distal part of optic vesicle) expresses *VSX2* while the proximal part that develops into RPE expresses *MITF*. The prospective optic stalk expresses *PAX2*. Boundary between RPE and neural retina is defined by the mutual antagonism between *MITF* and *VSX2*, while boundary between optic stalk and neural retina is defined by antagonism between *PAX2* and *PAX6* [4]. Several extrinsic factors including members of families of transforming growth factor (*TGF-β*), fibroblast growth factor, sonic hedgehog (*SHH*), and *WNT* signalling pathway modulate the effect of the intrinsic factors [5].

### Genetics and coloboma

Inherited cases of coloboma and those associated with chromosomal defects can be associated with systemic anomalies in addition to the ocular coloboma. The Online Mendelian Inheritance in Man web site catalogues the human genes related to genetic disorders with emphasis on genotype–phenotype correlation. Excellent articles by Gregory-Evans et al. [6] and Chang et al. [7] provide a comprehensive list of reported associations of coloboma with systemic syndromes as well as the identified gene loci or chromosome aberrations. In the current review article, information from these articles and from the cross references were evaluated and presented in Tables 1 and 2. Cases reported as choroidal coloboma or retinal coloboma have been grouped as chorio-retinal coloboma. The coloboma was sub-grouped as—isolated iris coloboma, isolated optic disc coloboma or chorio retinal coloboma (with or without iris or disc coloboma). The associations were also grouped based on inheritance pattern.

**Table 1:** Familial coloboma.

	Disease/abnormality (genetic locus)	Systemic associations
Autosomal recessive	***Isolated iris coloboma***	
	Congenital disorder of glycosylation type IV (3q27 (*ALG3*))	Microcephaly, dysmorphic face, tetraspastic palsy, psychomotor handicap
	Goldberg–Shprintzen megacolon syndrome (10q22.1)	Microcephaly, Hirschprung disease, megacolon
	Seckel syndrome 3 (14q24.3 (*SCKL*))	Microcephaly, mental retardation
	Meckel Gruber syndrome (17q22-q23 (*MKS1*))	Severe ciliopathy, can be lethal, occipital encephalocele, multiorgan defects
	Fronto facionasal dysostosis (unknown)	Encephalocele, hypertelorism, midface hypoplasia, frontal bone hypoplasia, deformed nostrils, cleft lip and palate
	Donnai Barrow syndrome (unknown)	Diaphragmatic hernia, hypertelorism, agenesis of corpus callosum, sensory neural deafness
	Kapur–Toriello syndrome (unknown)	Cleft lip, cleft palate, heart defects, distinctive nose
	Anterior chamber cleavage disorder (unknown)	Cerebellar hypoplasia, tracheal stenosis, hypothyroidism, dislocated hips
	***Chorio-retinal coloboma ± Iris coloboma ± optic disc coloboma***	
	Dihydropyrimidine dehydrogenase deficiency (1p22 (*DPD*))	Microcephaly, mental retardation, growth retardation, autism, epilepsy
	Warburg micro syndrome 1 (2q21.3 (*RAB3GAP*))	Mental retardation, dysmorphic face, hypo genitalism
	Joubert syndrome I (8q22.1 (*TMEM67*)). Coloboma seen in 80% with *TMEM67* mutation	Ciliopathy, brain stem and cerebellar vermis abnormality, extra ocular movement abnormality, ptosis
	Joubert syndrome type B (Cerebello oculo renal syndrome) (11p12-q13.3 (*CORS2*))	Above + cystic kidney
	Microphthalmia (14q24.3 (*CHX10*))	Isolated microphthalmia
	COACH syndrome (unknown)	Cerebellar vermis hypoplasia, oligophrenia, congenital ataxia, hepatic cirrhosis
	Temtamy syndromeq (unknown)	Macro dolichocephaly, hypertelorism, micrognathia, dental anomalies, absent corpus callosum, skeletal anomalies
	Ritscher–Schinzel syndrome (unknown)	Cleft palate, hypertelorism, low set ears, micrognathia, cardiac defects, cerebellar vermis hypoplasia, Dandy Walker malformation of brain
	Dubowitz syndrome (unknown)	Pre- and post-natal growth retardation, microcephaly, eczema, multisystem involvement.
	Chime syndrome (unknown)	Congenital heart disease, migratory ichthyosiform dermatosis, mental retardation, deafness
	Biemond syndrome type 2 (unknown)	Mental retardation, obesity, polydactyly, hypogonadism, hydrocephaly, facial dysostosis. Closely related clinically to Bardet–Biedel syndrome
	Chondrodysplasia punctata (unknown)	Radiological abnormalities of limbs, coarctation of aorta, hydronephrosis, partial absence of corpus callosum, brachy telephalangy of hands and feet
	***Isolated optic disc coloboma***	
	Walker–Walburg syndrome (9q34.1 (*POMT1*))	Absent corpus callosum, lissencephaly, hydrocephalus
Autosomal dominant	***Isolated iris coloboma***	
	Reiger’s syndrome type 1 (4q25-q26 (*PITX2*))	Sclero cornea, developmental glaucoma, dental anomalies
	Basal cell nevus syndrome (9q22.3 (*PTCH*))	Basal cell carcinoma, skeletal anomalies
	Retinal binding protein deficiency (10q24 (*RBP4*))	Non detectable scotopic electro retinogram
	Cataract and microcornea (16q22-q23 (*MAF*))	Anterior segment dysgenesis. No systemic disease
	Heterochromia iridis (unknown)	No systemic issues
	Pai syndrome (unknown	Midline cleft lip, midline facial polyps, lipoma of corpus callosum
	Scalp-ear-nipple (Finlay–Marks) syndrome (unknown)	Scalp defect, malformed ears, absent nipples, syndactyly, tooth and nail abnormalities
	Curry Jones syndrome (unknown)	Skull asymmetry, craniostenosis, pre-axial poly syndactyly, agenesis of corpus callosum, skin streaks
	***Chorio-retinal coloboma ± Iris coloboma ± optic disc coloboma***	
	Holoprosencephaly (2p21(*SIX3*))	Midline anomalies of brain
	Hirschsprung syndrome (2q22 (*ZFHX1B*))	Hirschsprung syndrome
	Congenital contractural arachnodactyly syndrome (Beal’s syndrome) (5q23-q31(*FBN2*))	Dysmorphism, arachnodactyly, kyphoscoliosis, joint contractures, blepharophimosis
	Treacher Collin syndrome (5q32-q33.1(*TCOF1*))	Deafness, downward sloping of palp fissure, lid coloboma, mandible and zygoma hypoplasia, cleft palate
	Uveal retinal coloboma (7q36 (*SHH*))	Non-syndromic
	Noonan syndrome (12q24.1(*PTPN11*))	Typical facial dysmorphic features: triangular facies, ptosis, epicanthal folds, low set ears, low hairline, webbed neck, widely spaced nipples, syndactyly of toes, cardiac abnormalities
	Oculo auriculo-vertebral dysplasia (14q32)	Microtia, hemifacial microsomia, epibulbar dermoids, vertebral anomalies, eyelid coloboma
	Colobomatous microphthalmos (15q12-q15)	Non syndromic
	Rubenstein Taybi syndrome (16p13.3(CREBBP))	Facial dysmorphism, polydactyly, skeletal and vertebral defects, Chiari type I malformations, renal malformations, endocrine disorders
	Townes Brocks syndrome (16q12.1 (SALL1))	Imperforate anus, polydactyly, triphalyngeal thumb, dysplastic ears, renal abnormalities, heart defects
	Oculo oto dental syndrome (20q13.1)	Sensory neural deafness, dental anomalies
	Acro-reno-ocular syndrome (20q13.13-q13.2(SALL4))	Polydactyly, hand and thumb anomalies, renal ectopia, vesiculo-ureteral reflux
	Steinfeld syndrome (unknown)	Cleft palate, holoprosencephaly, dysplastic ears, vertebral anomalies, heart defects, kidney malformations
	Macrophthalmia (unknown)	No systemic associations. Microcornea with increased axial length, myopia
	Dominant coloboma-microphthalmos with cleft lip (unknown)	Cleft lip and palate, sensory deafness, haematuria
	Kabuki syndrome (unknown)	Typical facies: long palpebral fissures, prominent eyelashes, arched eyebrows with lateral thinning, epicanthus, eversion of the lateral third of the lower eyelid, congenital heart defects, kidney defects
	MOMO syndrome (unknown)	Macrosomia, obesity, macrocephaly, ocular abnormalities
	***Isolated optic disc coloboma***	
	Renal coloboma syndrome (10q24.3(*PAX2*))	Vesiculo-ureteral reflux, small dysplastic kidneys
	Ocular coloboma and PAX6 mutations (11p13(*PAX6*))	Non syndromic
X-linked recessive	***Isolated iris coloboma***	
	Renpenning syndrome 1 (Xp11.23 (PQBP1))	Microcephaly, mental retardation, long narrow face, short stature
	Catel–Manzke syndrome (Xq21)	Hyperphalangy, micrognathia, malformed ears
	***Chorio-retinal coloboma ± Iris coloboma ± optic disc coloboma***	
	Lenz syndrome (Xp11.4(BCOR))	Mental retardation, palatal and dental anomalies, heart and renal defects
	***Isolated optic disc coloboma***	
	New X-linked syndrome with Corpus callosum defect (Xp13.1-q13.3(IGBP1))	Mental retardation, agenesis of corpus callosum, deafness, short stature, choanal atresia, cardiac defects
X-linked dominant	***Isolated iris coloboma***	
	Terminal osseous dysplasia and pigmentary defects (Xq27.3-q28)	Distal limb abnormalities, skin pigment defects, digital fibroma. Lethal in males.
	***Chorio-retinal coloboma ± Iris coloboma ± optic disc coloboma***	
	Oto palate digital syndrome (Xp22.31;Xq28 (FLNA))	Cerebellar hypoplasia, hydrocephalus, cleft palate, finger and toe abnormalities, bow legs, small jaw, sensory neural deafness
	Goltz focal dermal hypoplasia (Xp22.31)	Linear dermal atrophy and hyperpigmentation, extremity abnormalities such as syndactyly.
	***Isolated optic disc coloboma***	
	Aicardi syndrome (Xp22.31)	Agenesis of corpus callosum, asymmetry of hemispheres, periventricular heterotropia, cysts of choroidal plexus, infantile spasms

**Table 2:** Coloboma and chromosomal aberrations.

Type of aberration	Syndrome/location of chromosomal aberration	Coloboma	Systemic and other ocular associations
Triploidy	69; XXY chromosomes	Iris	Persistent hyperplastic primary vitreous, retinal dysplasia, cebocephaly, single midline nostril, syndactyly
Trisomy	Patau syndrome (Trisomy 13)	Iris	Microphthalmos, short survival, holoprosencephaly, cardiac and urogenital anomalies
	Trisomy 22	Chorio-retinal	Craniofacial anomalies
Deletions	16q syndrome (16q23.1-16q24.2 del)	Iris	Broad prominent forehead, large nose and mouth, short stature.
	Craniosynostosis (2q24.3 and 2q31 del)	Iris; chorio-retinal; optic disc	Craniosynostosis, mental retardation, syndactyly, campodactyly, small mandible, hypertelorism, proptosis
	Wolf–Hirschhorn syndrome (4p16.3 del)	Iris	Microcephaly, seizures, characteristic facies: high forehead, frontal bossing, hypertelorism, low set ears
	4q26 deletion syndrome (4q23-q27del)	Iris, chorio-retinal	Microphthalmos, prominent forehead, epicanthus, broad nose
	7q deletion syndrome (7q34-ter.del)	Chorio-retinal	Psychomotor retardation, dysmorphism
	Charge syndrome with micro deletion 8q12.1	Iris; chorio-retinal; optic disc	Cardiac defects, choanal atresia, growth retardation, genital hypoplasia, cleft palate, facial nerve palsy, deafness
	Jacobsen syndrome (11q23-q25 del)	Iris; chorio-retinal	Growth retardation, telecanthus, CNS abnormalities, endocardial cushion defect, trigonocephaly, facial dysmorphism, heart defects
	13q deletion syndrome (13-q13.2-ter del; 13q12-q32 del; 13q14-13q32 del)	Iris; chorio-retinal	Microphthalmos, holoprosencephaly, absent corpus callosum, skeletal anomalies, retinoblastoma described
	DiGeorge syndrome (22.11.2 del)	Iris; chorio-retinal	Microphthalmos, thalamic aplasia, heart defects, chronic infections, skeletal and renal anomalies, dysmorphism, hypotonia
Inversion	Baraitser–Winter syndrome (2p12-q14 inv)	Iris; chorio-retinal	Mental retardation, pachygyria and cortical atrophy
Translocation	Hypomelanosis of Ito. (Xp11.2 Mosaicism)	Iris; chorio-retinal	Incontinentia pigmenti achromians, hypomelanosis of ito, developmental delay, dental anomalies
	Coloboma with agenesis of corpus callosum (2p24 and 9q32)	Iris; chorio-retinal	Agenesis of corpus callosum, periventricular nodular heterotopia
Duplication	Cat eye syndrome (22q11)	Iris; chorio-retinal	Hypertelorism, pre-auricular skin tags or pits, anal anomalies, heart defects, down slanting palpebral fissure, micrognathia, renal, genital and skeletal anomalies, developmental delay/mental retardation

Screening for mutations in genes related to embryological factors connected with embryonal fissure has not been successful. The MAC study [8] failed to reveal any pathogenic mutations in *PAX6, CHX10,* and *SIX3* in patients with coloboma fundus. In another study of 30 patients of fundus coloboma, no mutations were identified in the *PAX6* gene [9].

Thus, the genetic associations can be summarised as follows:

(1) The genetic basis for occurrence of coloboma appears to be complex.

(2) Colobomas can occur in isolation or occur with systemic abnormalities.

(3) Colobomas associated with chromosomal aberrations are more likely to have systemic abnormalities.

(4) Multiple genes were identified in association with Coloboma. This is indicative of the complexity of the developmental process involved in the formation and closure of the choroidal fissure.

(5) Phenotype varies considerably and does not correlate with the genotype.

(6) Isolated coloboma (with no systemic abnormalities) can be sporadic, or familial (autosomal dominant, autosomal recessive or X-linked).

(7) Coloboma is associated with several syndromes, with ‘The coloboma of the eye, heart defects, atresia of the nasal choanae, retardation of growth, genital and/or urinary abnormalities, and ear abnormalities and deafness (CHARGE) syndrome’ being the commonest. While the gene for CHARGE syndrome is identified (*CHD7)*, the exact function of its gene product is not known.

(8) Embryologically, the following genes have been identified to be involved in the optic fissure formation and closure- *SHH* gene, *PAX2*, *PAX6*, *VAX* genes, etc. However not all patients with mutations in these genes develop coloboma and as seen from Table 1, there are many other genes that have been associated with coloboma.

#### Sporadic coloboma

Sporadic occurrence of coloboma could be

(a) Due to environmental factors causing intra-uterine insult.

(b) Genetic origin but having low penetrance or low expressivity.

(c) Genetic predisposition with environmental factor acting as a precipitating factor.

Evidence for environmental causative factors is mostly circumstantial. Several factors have been proposed including Vitamin A deficiency, maternal diabetes and hypothyroidism, maternal consumption of drugs such as thalidomide, carbamazepine, hydantoin, maternal alcoholism, etc. In experimental animals such as rats and mice, exposure during pregnancy to retinoic acid, saccharine, irradiation and folate deficiency has been shown to produce coloboma in the offspring.

### Systemic disorders and coloboma

Table 3 groups the reported systemic disorders into broad categories concerning various organ systems in the body. Fifteen to thirty percent of colobomas can have CHARGE syndrome. Shah et al. in a study in United Kingdom have shown the association with systemic features to be more common in bilateral cases of AMC (Anophthalmia, microphthalmos, coloboma) [10]. In a study by Huynh et al. of 99 patients with apparently isolated uveal coloboma, abnormalities were detected on echocardiography, renal ultrasound, audiology, X-ray of the spine and MRI of the brain [11]. Most of these, however, did not require urgent intervention. Faced with an apparently isolated uveal coloboma, they suggest a protocol including physical examination, baseline audiology assessment, kidney ultrasound and spine X-ray [11]. Table 4 explains some of the terms related to systemic abnormalities associated with fundus coloboma that may be unfamiliar to an ophthalmologist.

**Table 3:** Systemic associations with coloboma.

S no.	Region/system with abnormality	Described associations
1	Peri ocular	Ptosis, hypertelorism, blepharophimosis, eversion of lateral third of lower eyelid, eyelid coloboma, epicanthal folds, epibulbar dermoids, arched eyebrows, prominent eyelashes
2	Facial	Midface hypoplasia, frontal bone hypoplasia,, micrognathia, dental abnormalities, triangular facies, mandible and zygoma hypoplasia, webbed neck, low hairline, macrostomia, cleft lip and palate
3	Cephalic	Macrocephaly, microcephaly, encephalocele, macro dolichocephaly, hydrocephaly, facial dysostosis (e.g. Treacher Collins syndrome), craniostenosis, trigonocephaly, cebocephaly
4	Central nervous system	Holoprosencephaly, agenesis of corpus callosum, lipoma of corpus callosum, cerebellar hypoplasia, cerebellar vermis abnormality (Dandy Walker malformations of brain), lissencephaly, pachygyria, asymmetry of hemispheres, seizures, ataxia
5	Ear, nose and throat	Low set ears, microtia, anotia, deformed nostrils, choanal atresia, sensory neural deafness
6	Spinal	Kyphoscoliosis, Chiari type 1 malformations.
7	Limbs	Dislocated hips, joint contractures, polydactyly—preaxial or post axial, telephalangy, arachnodactyly, syndactyly, campodactyly, hyperphalangy, bow legs
8	Cardio vascular system	Congenital heart defects, coarctation of aorta
9	Respiratory system	Tracheal stenosis
10	Renal	Cystic kidney, hydronephrosis, renal ectopia
11	Genito urinary	Vesiculo-ureteral reflux, hypogonadism
12	Gastro intestinal	Hirschprung disease, megacolon, imperforate anus
13	Cutaneous	Eczema, migratory ichthyosiform dermatosis, linear dermal atrophy, skin pigmentation
14	General	Growth retardation, mental retardation, obesity, large size of baby

**Table 4:** Glossary of terms related to systemic abnormalities associated with fundus coloboma that may be unfamiliar to an ophthalmologist.

1	Agenesis of corpus callosum	Partial or complete absence of development of corpus callosum that connects the two cerebral hemispheres
2	Anotia	Absent external ear
3	Arachnodactyly	Abnormally long slender fingers
4	Campodactyly	One or more fingers are permanently bent
5	Cebocephaly	Part of spectrum of holoprosencephaly, single nostril with no septum, two separate eyes but close together
6	Chiari type 1 malformations	Protrusion of brain tissue into spinal canal. Type 1 occurs as the growth of brain and skull takes place and hence is acquired, compared to types 2 and 3 that are congenital and more severe.
7	Choanal atresia	Narrowing or blockage of the back of the nasal cavity by soft tissue or bone-causing difficulty in breathing.
8	Coarcation of aorta	Narrowing of part of the aorta
9	Craniostenosis	Premature closure of one or more joints between bones of the skull.
10	Dandy Walker malformation of brain	Partial or complete non-development of cerebellar vermis that connects the two cerebellar hemispheres.
11	Dysmorphism	A generic term to indicate abnormal body structure including malformation (abnormal development), dysplasia (abnormal growth or organisation within a tissue), deformation (damage caused by outside force).
12	Encephalocele	Sac-like protrusion of brain and the membranes through openings in skull
13	Hemifacial microsomia	Abnormal smallness of one half of face (microsomia means abnormally small body structures)
14	Holoprosencephaly	A disorder where the prosencephalon that develops into the forebrain fails to develop into two hemispheres.
15	Hirschprung disease	A type of congenital megacolon caused by absence of ganglionic cells
16	Hyperphalangy	Having more than normal number of phalanges in a finger.
17	Incontinentia pigmenti achromians (hypomelanosis of ito)	Patterns of bilateral or unilateral hypopigmentation following the lines of Blaschko. Lines of Blaschko are lines of normal cell development in skin (embryonic cell migration) that are apparent only in disease—e.g. ‘V’ shape on back, ‘S’-shaped whirls on chest and sides, wavy shapes on head, etc.
18	Kyphoscoliosis	Abnormal curvature of spine in two planes—side to side and back to front
19	Lissencephaly	Absence/ reduction of normal convolutions in the brain.
20	Macrocephaly	Size of head larger than 2 standard deviations from normal for the age and sex. Can be caused by hydrocephalus or megalencephaly (enlarged brain) secondary to metabolic disorders, neurofibromatosis, etc.
21	Macro dolichocephaly	Where the skull is longer than expected relative to its width.
22	Macrostomia	Unusually wide mouth
23	Macrosomia	Larger than expected size of baby at birth.
24	Megacolon	Abnormal dilation of colon not caused by obstruction
25	Microcephaly	Size of head smaller than two standard deviations from normal for the age and sex.
26	Micrognathia	Small lower jaw
27	Microtia	Small external ear
28	Migratory ichthyosiform dermatosis	Sharply demarcated hyperkeratotic plaques that migrate.
29	Oligophrenia	Less than normal mental development
30	Pachygyria	Reduced, broader and flat gyri in the brain.
31	Post axial polydactyly	Extra finger next to little finger (Ulnar) or extra toe next to little toe (lateral)
32	Pre-axial polydactyly	Extra finger next to thumb (radial) or extra toe next to greater toe (medial)
33	Syndactyly	Fusion of soft tissues of two adjacent fingers with or without bone fusion
34	Telephalangy	Shortening of distal phalanges with cone shaped epiphysis
35	Tetraspastic palsy	All four limbs involved in spastic paralysis.
36	Trigonocephaly	Premature fusion of the metopic suture between two frontal bones leading to restricted transverse growth and producing triangular skull

### Presenting symptoms

(1) Anomaly noticed by parents/paediatrician: the majority of cases are identified because of an anomalous looking eye. This visible anomaly could be due to obviously small eyeball, gross nystagmus, strabismus or an obvious iris coloboma.

(2) Chronically poor vision: coloboma even without superadded RD can cause chronically poor vision due to uncorrected refractive error, amblyopia, involvement of fovea, etc. These defects surface at time of initiation of schooling if they have not been already detected.

(3) Acute reduction in vision with or without prior chronic poor vision: acute onset RD is the commonest cause of acute loss of vision in an eye with coloboma. Other causes of acute reduction in vision can be sub foveal choroidal neovascular membrane. In children, often the visual disability is picked up when both eyes are affected—with one eye having long-standing RD and the other having relatively recent RD. It is also difficult for the parents to note reduction in vision on top of an already existing poor vision.

(4) Asymptomatic: patients with small colobomata that do not involve disc and fovea can remain asymptomatic unless complicated by RD.

(5) Leukocoria: large coloboma can produce a yellow/white pupillary reflex. A complicated cataract can also draw the attention of the parent to an issue in a child’s eye.

### Clinical features

The involvement can be unilateral (33–47.5% of cases) or bilateral and when bilateral can be symmetric or asymmetric [12].

#### Nystagmus

If central fixation is affected in both eyes due to macular involvement, nystagmus can be the presenting symptom. Often these eyes are also significantly microphthalmic.

#### Anterior vs. posterior segment involvement

The study by Nakamura has shown isolated anterior segment involvement (iris and ciliary body) in 36%; isolated posterior segment involvement (chorio retinal and optic nerve) in 39% and involvement of both in 24% of cases [13].

#### Microphthalmos and microcornea

It is important to understand that corneal diameter cannot always serve as surrogate marker of size of the eye. While eyes with microphthalmos have correspondingly small corneas, microcornea can exist with normal sized eyeballs [14, 15]. Accurate assessment of eye-ball size is possible with ultrasonography, CT scan or MRI. By definition, an eye is labelled microphthalmic, when the axial diameter (adjusted for age) is <95th percentile [16]. In adults (and children >13 years age), axial diameter <18.5 mm is considered microphthalmic.

#### Caveat

Considering the often coexisting ectasia of the colobomatous area, the measurement on ultrasonography can be falsely normal/more than normal, if measurements are taken within the ectatic area. This is important in eyes with extensive coloboma involving significant part of the posterior pole.

#### Microphthalmos with orbital cyst

During development, defective closure of embryonal fissure results when neuroectodermal hyperplasia leads to eversion of the inner layer at the edge of the embryonal fissure. If the neuroectoderm has not yet differentiated into retina, the fissure may still close, resulting in ectatic coloboma with ICM. If, however, retinal differentiation has occurred, fusion does not take place leading to formation of true orbital cyst [1]. The size of the cyst can be variable and can sometimes reach such large proportions to be mistaken for an orbital tumour. The eyeball, in these extreme cases, is severely microphthalmic or hardly recognizable clinically—having been pushed aside or posteriorly by the cyst. The size of communication between the cyst and the vitreous cavity is variable. In most such cases, the eye is non-functional with the retina being dysplastic and the management is directed towards better cosmesis [17, 18].

#### Iris coloboma

**Fig. 1:**
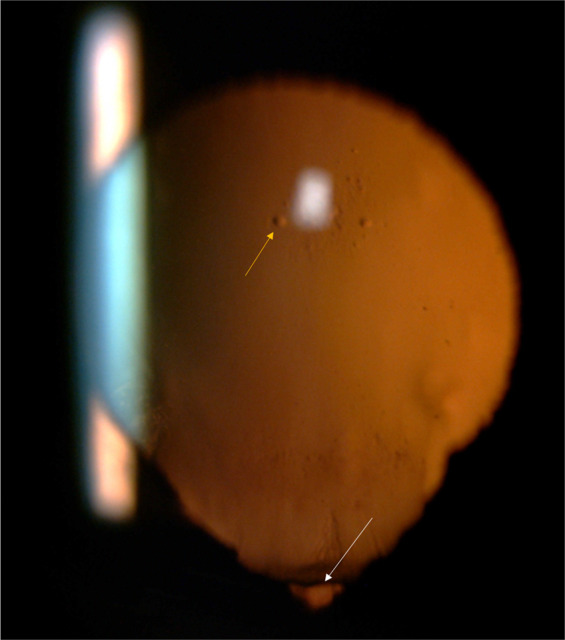
Slit-lamp photograph demonstrating complete iris coloboma. Note (i) The visibility of equator of the lens inferiorly with possibly no zonules in this area (white arrow). (ii) Early vacuoles in an otherwise clear lens (yellow arrow) (color figure online).

**Fig. 2:**
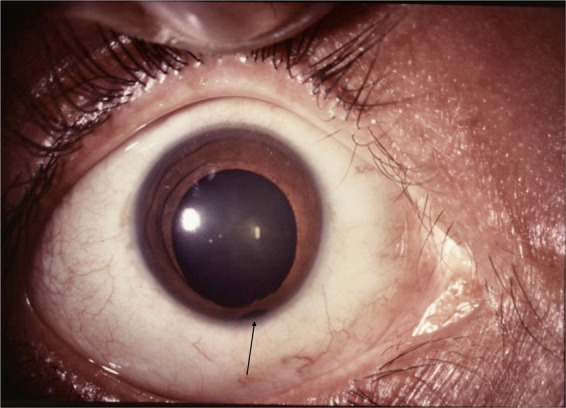
Anterior segment photograph demonstrating incomplete iris coloboma. Observe the notch in inferior pupillary border and partial thickness iris defect near iris root (arrow).

Iris is commonly involved in eyes with fundus coloboma but this association is not compulsory and does not correlate with the severity of fundus coloboma [19]. Complete iris coloboma is seen as a defect infero nasally that merges with pupil (key hole iris) (Fig. 1), while partial iris coloboma can be seen as a notch in the sphincter, defect in pigment epithelium or heterochromia (Fig. 2) [20]. Unlike traumatic iris defects, the margins of a coloboma are smooth.

#### Ciliary body coloboma

In the region of the coloboma, ciliary muscle and pigment epithelium are absent. Coloboma of ciliary body is often associated with coloboma of iris. The zonules are absent in this area and the lens equator is deformed as a consequence.

#### Choroidal coloboma

The visually significant part of the colobomatous defect is the fundus coloboma. The descriptions and classifications of severity revolve around the relationship of coloboma with optic disc and macula. Choroid and RPE are absent in the area of coloboma. The sclera in the floor of the coloboma is thinned out to a variable degree and this can result in ectasia of the coloboma. The internal surface has bare sclera with occasional splash of pigment near the border and can be smooth or have scalloped surface. Antero posteriorly the coloboma can reach and break out into the periphery or be restricted to island (s) along a line joining disc with inferior/infero-nasal periphery. The transverse reach of the coloboma usually varies directly with the anteroposterior extent. Transversely, the largest colobomas can almost fill the entire inferior fundus. The severity of disc involvement is also more in such large colobomata (see below) [19]. Bridge coloboma is a term used to describe two islands of colobomas with normal retina in between.

### The optic disc and the choroidal coloboma

The involvement of the optic disc in the colobomatous process is complex. Classifications of choroidal coloboma mostly revolve around the optic disc involvement—both in terms of physical location within or outside the choroidal coloboma as well as whether the optic disc itself is involved in the colobomatous process.

#### Classification

**Fig. 3:**
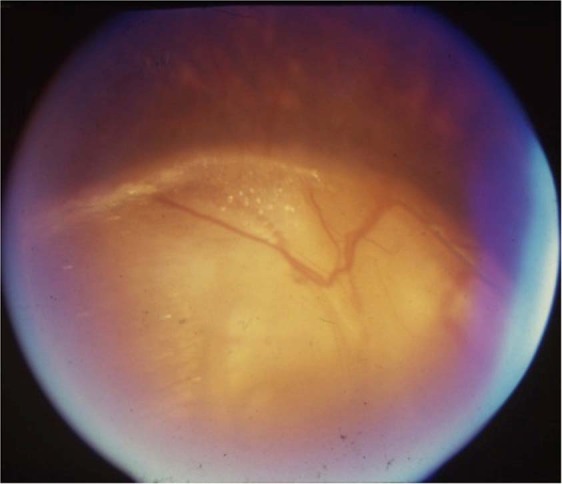
Fundus photograph of choroidal coloboma. (i) The coloboma extends above the optic disc (type 1 of Ida Mann classification). (ii) Disc coloboma merges with choroidal coloboma with disc substance hardly made out (type 6 in classification by Gopal et al.).

**Fig. 4:**
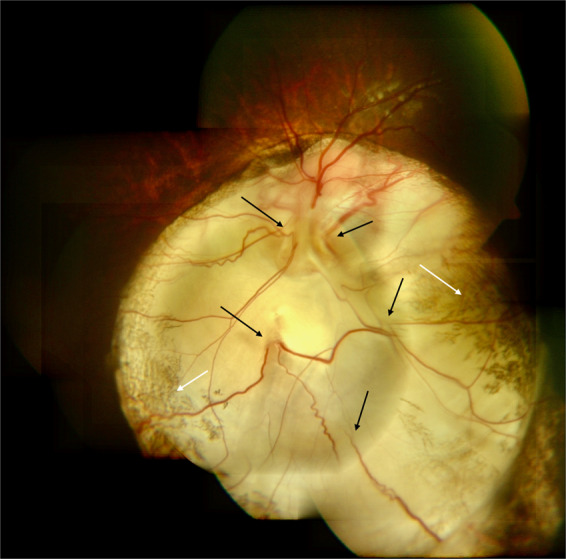
Fundus photograph of choroidal coloboma. (i) The fundus coloboma just skirts the upper disc border (type 2 of Ida Mann classification). (ii) Disc coloboma merges with fundus coloboma but upper part of disc is well delineated (type 5 of classification by Gopal et al.) (iii) Vessels meant for upper fundus emanate from lower part of the identifiable disc and smoothly go upwards across the disc. (iv) Multiple sites of emergence of inferior blood vessels from bed of the coloboma (black arrows). (v) Splash of pigment near periphery of the coloboma although there is no definable choroid or RPE (white arrows).

**Fig. 5:**
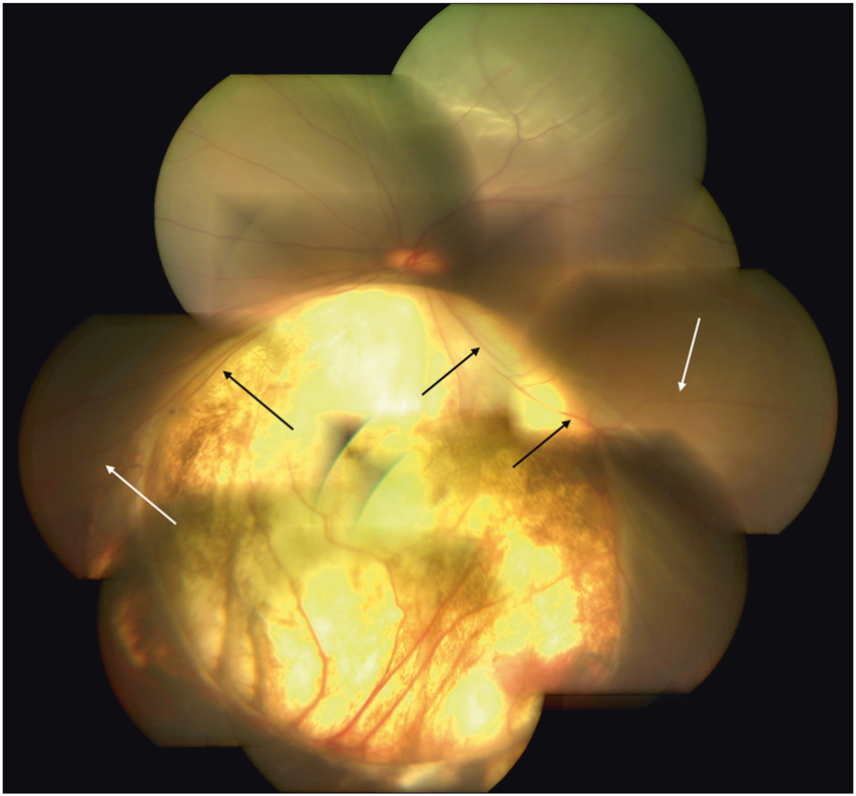
Fundus photograph of choroidal coloboma. (i) Coloboma reaches up to lower border of disc (type 3 of Ida Mann classification). (ii) The disc is outside the coloboma and apparently normal (type 1 of the classification by Gopal et al.). However, observe the flattening of inferior border of disc. (iii) Inferior retinal blood vessels traversing within the coloboma near the border (black arrows) before supplying infero-nasal and infero temporal peripheral retina (white arrows).

**Fig. 6:**
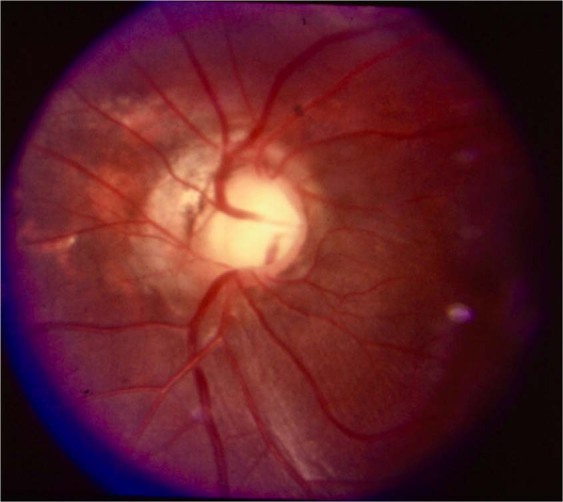
Fundus photograph of isolated disc coloboma (type 4 Ida Mann classification). Notice the concentric coloboma that mimics glaucomatous cupping and some degree of peripapillary atrophy.

**Fig. 7:**
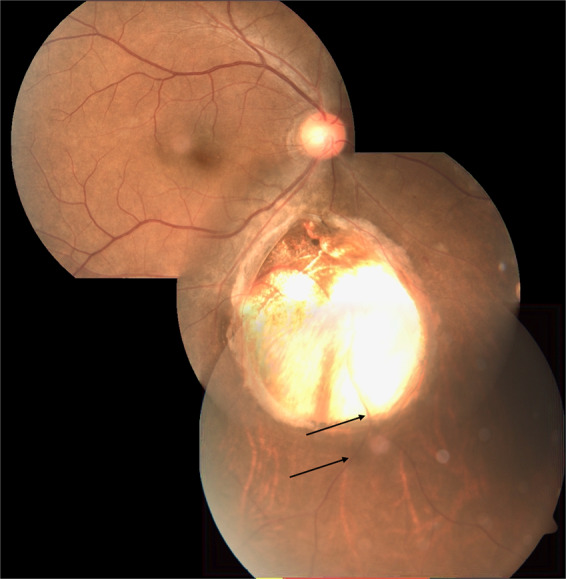
Fundus photograph of choroidal coloboma. (i) Coloboma does not reach the disc and there is normal retina above and below disc (type 5 of Ida Mann classification). (ii) Normal disc located outside the coloboma (type 1 in classification by Gopal et al). (iii) The inferior retinal blood vessel traversing the middle of the coloboma is partly obscured—hidden within the sclera (black arrows).

**Fig. 8:**
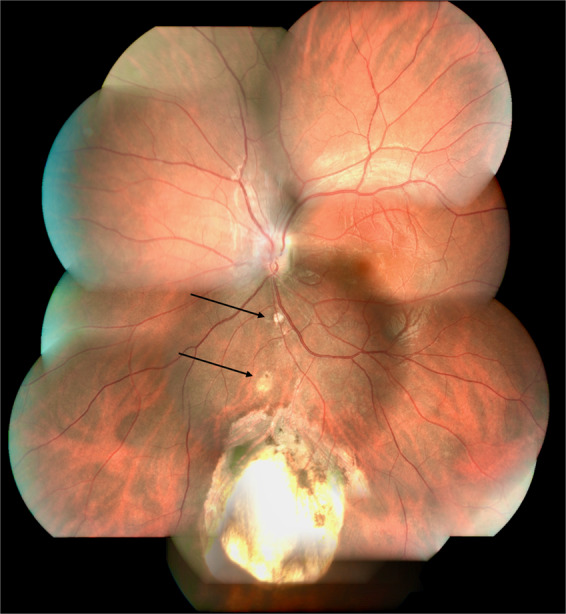
Fundus photograph of choroidal coloboma. (i) Small coloboma restricted to inferior periphery (type 7 Ida Mann classification). (ii) Disc located outside the coloboma but dysplastic (type 2 in classification by Gopal et al). (iii) Two additional patches of non-descript chorio retinal atrophy along the line joining the disc with the coloboma-possible forme fruste lesions (black arrows).

Ida Mann’s classification [21]: type 1—coloboma extending above the optic disc (Fig. 3); type 2—coloboma extending up to superior border of disc (Fig. 4); type 3—coloboma extending below the lower border of disc (Fig. 5); type 4—coloboma involving the disc only (Fig. 6); type 5—coloboma present below the disc with normal retina above and below the coloboma (Fig. 7); type 6—pigmentation present in the periphery; type 7—coloboma involving only the periphery (Fig. 8). In general, the disease severity is depicted from the most severe (type 1) to the least (types 6 and 7). A type 1 anomaly is expected to have the worst vision, while the type 7 anomaly is expected to have normal vision. Type 4 anomaly addresses isolated disc coloboma and type 5 anomaly represents a small coloboma in mid fundus with retina being normal anteriorly as well as posteriorly. The lacuna in this classification is that the type of disc involvement within the choroidal coloboma has not been specified.

**Fig. 9:**
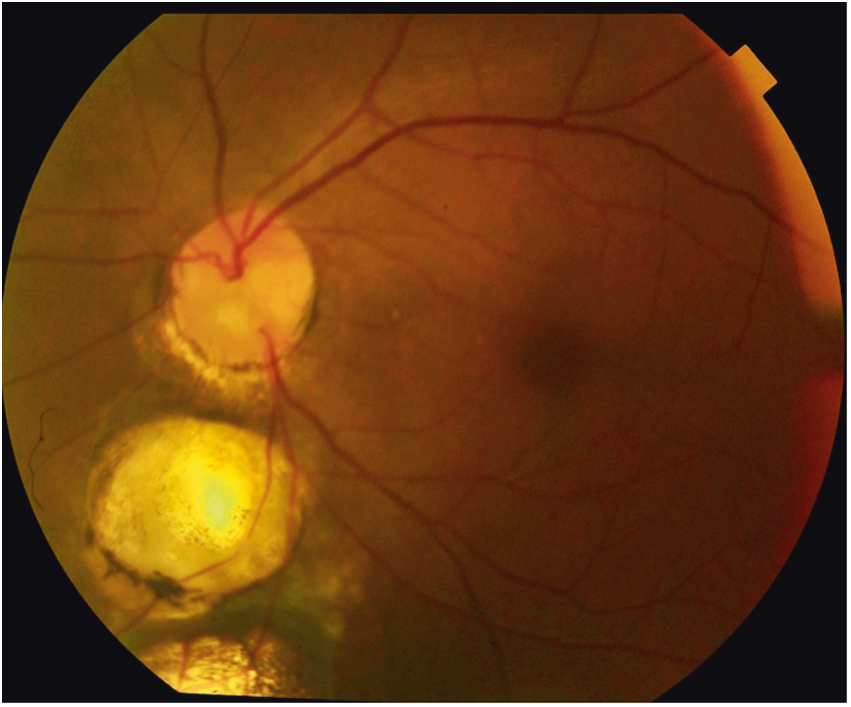
Fundus photograph with choroidal coloboma. (i) Two patches of coloboma below the optic disc and clearly separated from it. (ii) The disc is independently colobomatous with the coloboma occupying the inferior part of the disc (type 3 in classification by Gopal et al.).

**Fig. 10:**
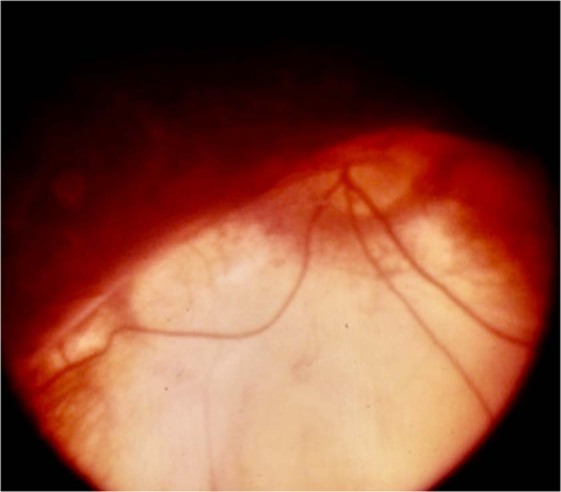
Fundus photograph with choroidal coloboma. Normal looking optic disc but located within the fundus coloboma (type 4 in classification by Gopal et al. and type 2 in classification by Ida Mann).

The classification by Gopal et al. divides these eyes into six types based on location and involvement of disc in the coloboma [19]. In types 1–3, the choroidal coloboma does not reach the optic disc. Type 1 has normal looking optic disc (Figs. 5 and 7). However, a flattening of inferior border is not uncommon (Fig. 5). Type 2 has dysplastic looking optic disc (Fig. 8) while type 3 has a disc that is independently colobomatous but located outside the choroidal coloboma (Fig. 9). Two types of disc coloboma were noted in type 3—an eccentric coloboma or one that resembled glaucomatous cupping. Types 4–6 have the disc enclosed in the choroidal coloboma. Type 4 has a normal looking optic disc surrounded by choroidal coloboma (Fig. 10), while types 5 and 6 have coloboma of the optic disc merging with the choroidal coloboma. Type 6 represents the most severe anomaly where in the disc substance is hardly made out (Fig. 3). Type 5 is the commonest configuration seen and the disc substance is made out in the upper part of the disc coloboma as a creamy pinkish area (Fig. 4). Large choroidal colobomas and poor vision were more often seen with types 4–6. The lacuna in this classification is that isolated coloboma at optic nerve entrance has not been considered.

### The transition zone between coloboma and normal fundus (Fig. 11)

**Fig. 11:**
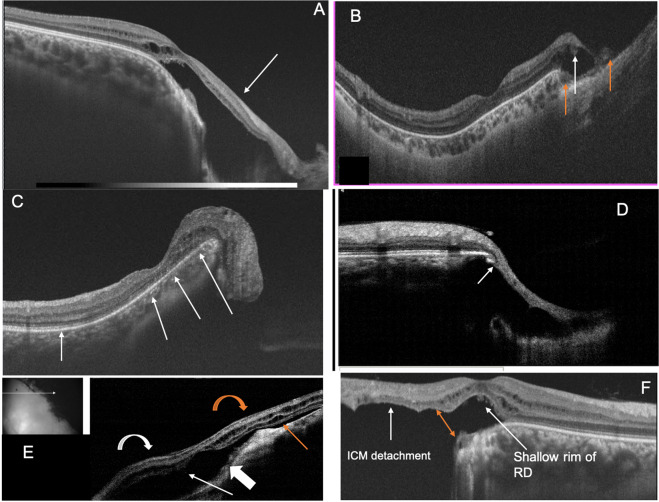
Collage of optical coherence tomography (OCT) images of transition zone. **A** OCT demonstrating gradual transition of retina to ICM (arrow). Also notice the detachment of ICM with extension into normal retina beyond coloboma margin and the detached macula located just beyond the coloboma margin. **B** OCT demonstrating abrupt transition from retina to ICM (white arrow). Also observe the sudden thinning and subsequent absence of choroid at the margin of coloboma (orange arrows). **C** OCT demonstrating the sharp upturn of the coloboma margin (white arrows). Notice the macula close to coloboma margin. **D** OCT demonstrating the location of Locus minoris resistantiae (arrow). Observe the outer retinal layers turning back to merge with RPE (white arrow). **E** OCT demonstrating retinal detachment (orange curved arrow) extending into ICM detachment (white curved arrow). The sub-ICM space (white arrow) continues into sub-retinal space (orange arrow) due to break in Locus minoris resistantiae (thick white arrow). Inset shows location of the OCT scan. **F** OCT demonstrating ICM detachment with shallow retinal detachment near coloboma margin and break in Locus minoris resistantiae (orange double arrow). Observe the detached macula just beyond the coloboma margin (color figure online).

This zone holds significant features that influence the occurrence of RD. Histologically, near the coloboma margin, the terminated RPE is adherent to the outer retinal layers. Beyond this point, the retinal layers transition into the non-descript fibrotic tissue called inter-calary membrane (ICM). This zone of adhesion is the point of least resistance termed ‘Locus minoris resistantiae’ [22]. The photoreceptor layers have been seen sometimes to turn back and merge with the terminated RPE, leading to a double layer of photoreceptors [22]. Figure 11D demonstrates the site of Locus minoris resistantiae.

The transition from the retina to the ICM can be gradual or abrupt (Fig. 11A, B). If the coloboma is sharply ectatic, the edge protrudes like an overhanging margin and this can be seen to turn inwards (Fig. 11C). The ICM can be taut exerting traction on the retina in the transition zone resulting in shallow RD beyond the coloboma—best appreciated on optical coherence tomography (OCT) (Fig. 11F) [23]. The area just beyond the visible coloboma border can have variable pigmentation and chorio retinal atrophy.

In eyes with RD caused by coloboma, ICM detachment of variable degree can be made out (Fig. 11E). Identification of even a rim of ICM detachment indicates the contribution of coloboma to the causation of RD. In the presence of ICM detachment, OCT can detect dehiscence in zone of least resistance—seen as communications between sub-retinal space and sub-ICM space (Fig. 11E, F).

### Blood vessels in the coloboma

Casper (quoted by Duke Elder) described three patterns of emergence of blood vessels from the disc. (i) The most common arrangement was the emergence of blood vessels from the lower part of the colobomatous disc. The blood vessels meant for the inferior fundus make a sharp bend, kinking over the edge before proceeding inferiorly while vessels superior vessels progress directly across the excavated portion of the colobomatous disc. (ii) A normal looking arrangement of blood vessels emanating from approximately the centre of the colobomatous disc. (iii) Blood vessels emanating from the edge of the colobomatous disc resembling cilio-retinal vessels.

**Fig. 12:**
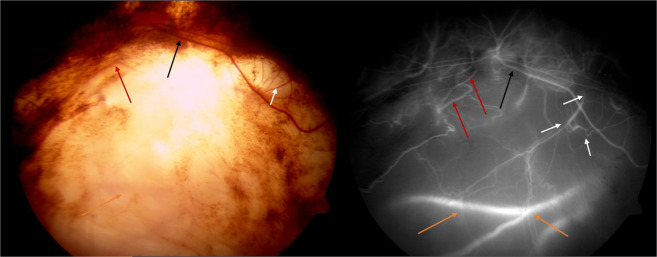
Fundus photograph and ICG angiogram demonstrating the type of blood vessels noted in the coloboma. (i) Disc just outside visible limit of choroidal coloboma—normal looking but with flattened inferior border. (ii) ICG shows absence of chorio capillaris within coloboma. (iii) Black arrows delineate the retinal blood vessel that emanates from the disc, traverses through the coloboma and enters normal infero temporal retina. (iv) Absence of branches while vessel is in coloboma. (v) White arrows delineate retinal vessels emanating from bed of coloboma with some proceeding towards temporal retina. On fundus photo these can be confused as branches of the vessel marked with black arrow but ICG clearly shows them as independent vessels. Also only parts of these vessels are seen in fundus photo. (vi) Red arrows delineate retinal blood vessel that can be traced on ICG to the disc although not so obvious on fundus photo. (vii) Orange arrows delineate probably extra ocular episcleral vessels (color figure online).

Studies correlating angiography with fundus photos have delineated four varieties of blood vessels in the coloboma (Fig. 12) [24].

(1) Retinal blood vessels that are continuous with the vessels emanating from optic disc. (2) Retinal blood vessels emanating from bed of coloboma but whose continuity with central retinal artery or its branches can be established, although not obvious. (3) Retinal blood vessels emanating from bed of coloboma that are possibly cilio-retinal vessels. (4) Broad deep vessels seen deep in the sclera, which are presumed to be extra ocular vessels that are visible through the thinned sclera.

ICG angiography has shown total absence of choroio-capillaris in the area of coloboma as well as in the adjoining chorio retinal atrophic patches. The blood vessels supplying the extra colobomatous normal retina could traverse in the normal retina or cross the coloboma before reaching the normal retina (Fig. 5). Within the coloboma, the entire blood vessel may be visible in the ICM or may be partly hidden in the ectatic sclera (Fig. 7) [24]. While in the ICM, the retinal vessels give out only few branches and resume normal branching after crossing the coloboma.

### Inter-calary membrane breaks

When ICM is detached, breaks can sometimes be made out within the ICM despite the lack of contrast due to absence of RPE and choroid. Three types of ICM breaks have been described [25].

**Fig. 13:**
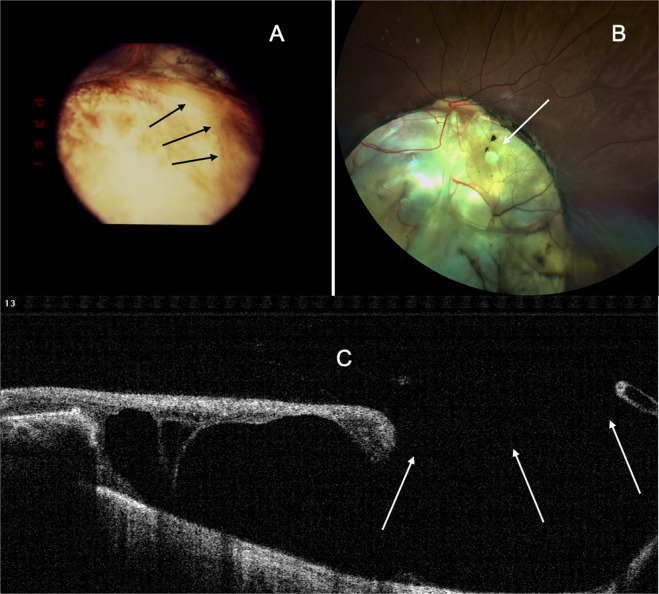
Collage of photographs demonstrating breaks in inter-calary membrane (ICM). **A** Fundus photo showing crescentic break in ICM (arrows). ICM peripheral to the break is lifted, while centrally ICM merges with the floor of coloboma. **B** Fundus photo showing a round hole in the anatomical macula that is involved in the coloboma (arrow). **C** OCT demonstrating the break in ICM (arrows) and ICM detachment. All edges of the break are lifted up.

(1) One or more oval breaks with all the edges lifted up (Fig. 13C). (2) Crescentic break along the border of coloboma where in only the ICM peripheral to the break is lifted up (Fig. 13A). (3) Breaks in the macular area where macula is involved in the coloboma (Fig. 13B).

### Forme fruste lesions

Manifestations of incomplete colobomas are not uncommon. In the iris, a notch may be seen in the sphincter infero-nasally or transillumination may reveal pigment defect. In the fundus, one may see posterior dipping of the ora serrata in the infero-nasal quadrant or patches of chorio retinal atrophy or RPE defects along a line connecting the disc to infero-nasal periphery (Fig. 8). By themselves these lesions are not diagnostic but coupled with other more obvious features of coloboma, their origin can be inferred. OCT has shown these fundus lesions to be mildly ectatic with retinal architecture relatively preserved [23]. In contrast, a small coloboma would have a moderately high reflective membrane with no identifiable retinal architecture.

### Congenital cavitary optic disc anomaly (CODA)

It is beyond the scope of this article to discuss in detail the various entities listed under CODA. Only a brief account of each one of them is given to permit better understanding of their association with embryonal closure defects.

Isolated (unaccompanied by choroidal coloboma) optic disc cavitary anomalies are a mixture of conditions—some definitely linked to defective embryonal fissure closure, some definitely not and some of unsure origin [26–28]. These include optic disc coloboma, morning glory syndrome, optic pit, peripapillary staphyloma and Pedler’s coloboma. Literature is confusing since terms have been used interchangeably although they mean entirely different entities [28, 29].

#### Condition definitely related to defective closure of embryonal fissure

##### Typical optic disc coloboma

Even in the absence of choroidal coloboma, optic disc coloboma is easy to recognize ophthalmoscopically—with bowl-shaped excavation, sharp borders and occupying the lower part of the disc. However, colobomas that mimic glaucomatous cupping have also been seen (Fig. 6) [19, 30]. There is no central glial tuft (unlike morning glory syndrome) and the vasculature on disc is essentially normal but for the displacement caused by the coloboma. It is caused by defective closure of the proximal part of the embryonal fissure.

#### Condition not related to defective closure of embryonal fissure

##### Peripapillary staphyloma

**Fig. 14:**
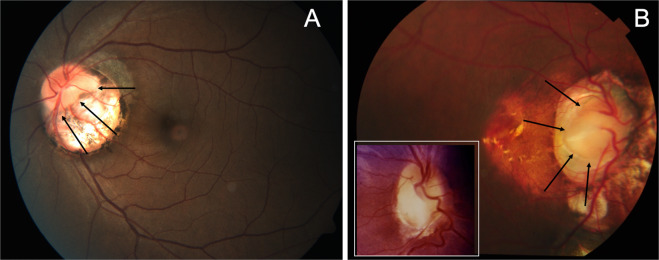
Fundus photographs demonstrating peripapillary staphyloma. **A** Normal disc is seen surrounded by peripapillary staphyloma. **B** The optic disc in this can be mistaken to be colobomatous. But close inspection clearly shows the disc to be normal (outlined by arrows). The inset shows typical optic disc coloboma for comparison.

A normal looking optic disc is seen at bottom of the excavation caused by outward bulging of eyewall (Fig. 14A, B). While Fig. 14A is typical, B can be mistaken to be optic disc coloboma with staphyloma. However, close inspection reveals that the disc is normal (compare with the disc coloboma in inset). Pigmentary changes are often seen surrounding the excavation and along its walls. Reports have also indicated contractile nature of the staphyloma in some cases [31]. Incomplete differentiation of the sclera from the neural crest and presence of a large scleral canal are hypothesised to cause reduced peripapillary scleral support [29]. Swept source OCT studies reveal a deep excavation with no glial tissue or membrane in the excavation. The retina beyond the excavation was found to be normal [32].

#### Conditions of unsure origin

##### Morning glory syndrome

**Fig. 15:**
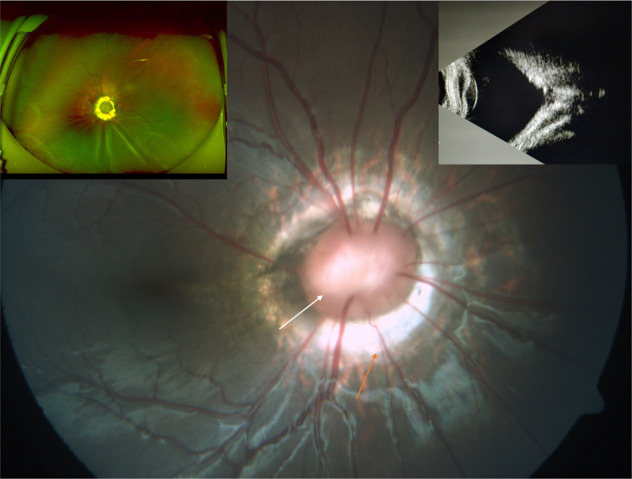
Morning glory syndrome. (i) Typical appearance of the optic disc with tuft of glial tissue at centre (white arrow), peripapillary RPE atrophy (orange arrow) and relatively straight retinal blood vessels radiating from the optic disc. (ii) Left inset shows a case of retinal detachment in an eye with morning glory syndrome. Observe the radiating folds caused by the puckering of the retina towards the disc. (iii) Right inset shows picture of ultrasonography demonstrating the funnel-shaped posterior excavation.

Ophthalmoscopically morning glory syndrome has a distinctive appearance (Fig. 15) [33]. It has a funnel-shaped excavation of the fundus in which the optic disc is situated (in contrast to excavation within the optic disc in optic disc coloboma). The posterior pole has pigmentary changes and atrophic patches around the excavation. A white tuft of glial tissue lies at the centre, which appears to be pulling the retina inwards into the slope. The retinal vasculature is abnormal with more than normal number of vessels arising out of disc and running a straighter course [29]. OCT studies have shown a well-defined pre-retinal membrane adherent to vitreous and pulling the retina centripetally as well as preventing excavation of the retina, unless the situation is altered by occurrence of posterior vitreous detachment (PVD) or surgical removal of vitreous [32]. Secondary RDs are common and can result in significant visual loss. Rhegmatogenous RDs result from slit-like breaks in the retina overlying the disc [34]. The condition is mostly unilateral with a wide range of presenting visual acuities and is more common in females. Systemic association with transsphenoidal encephalocele has been reported.

#### Pedler’s coloboma [35–37]

The unique feature of the reported cases appears to be a mass-like affect caused by traction of the peripapillary retina onto the disc area accompanied by scleral weakening and ectasia. This is hypothesised to be caused by defective scleral maturation. The reported cases were confused with optic disc neoplasm and were enucleated. The histo-pathological studies have shown this mass-like tissue to be gliotic retinal tissue and a thick retinal fold that has pushed the cribriform plate and optic disc posteriorly. The choroid was found to be prolapsing through the coloboma into the optic nerve sheath.

#### Optic disc pit

There is controversy as to the relationship between optic disc pit and defective closure of embryonal fissure. The evidence in favour is that optic pits often coexist with choroidal colobomas. Brown and Augsburger reported cases of optic pits (in nasal part of disc) in eyes with retino-choroidal colobomas [38]. Singerman and Mittra described a family of three generations with optic pits and iris coloboma [39]. Gopal et al. described a case with typical temporal optic pit in one eye along with forme fruste lesions in inferior fundus while fellow eye had typical retino-choroidal coloboma [23]. The predominantly temporal location of the optic pits (not infero-nasal) militates against this hypothesis.

Serous detachments of the macula and intra-retinal fluid are a common complication of optic pit and can cause long-term poor vision in these eyes [40]. Both vitreous and cerebro-spinal fluid have been proposed as the source of sub-retinal and intra-retinal fluid [38, 41, 42]. Briefly, the management options include observation, laser to temporal border of optic disc, vitrectomy and fluid air exchange [43], attempts to close the optic pit with internal limiting membrane flap, inner retina fenestration, etc. [44].

### Macular coloboma

**Fig. 16:**
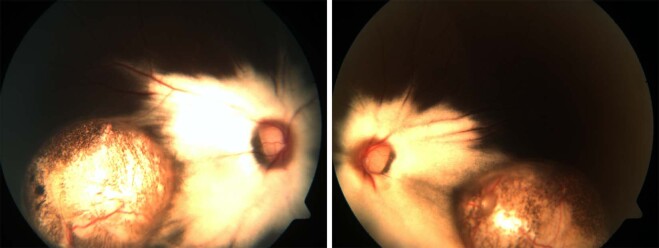
Fundus photo of macular coloboma. Observe well delineated atrophic patch of coloboma with some pigmentation near periphery. Also observe the dense patch of medullated nerve fibres all round the optic disc.

A coloboma in the macular area is believed to be not related to anomalous embryonal fissure closure. Macular coloboma is seen as oval or circular area of chorio retinal atrophy of variable size. The white sclera is seen in the centre with variable pigmentation at the border. Figure 16 shows a case of bilateral macular coloboma with medullated nerve fibres. Macular colobomata have been described as genetic disorders [45], associated with congenital amaurosis of Leber and other retinal dystrophies [46], and congenital toxoplasmosis. On OCT, congenital developmental colobomas have total absence of retina and choroid in the atrophic central area of the scar while in congenital toxoplasmosis, there is retinal thinning, RPE hyper-reflectivity, excavation, intra-retinal cysts and fibrosis [47, 48].

### RD and choroidal coloboma

The most important complication of choroidal coloboma is occurrence of RD. Although the actual prevalence of RDs related to chorio-retinal coloboma in the population is not known, hospital-based studies have reported a prevalence ranging from 2.4 to 47.5% [8, 12, 13, 49, 50]. It has been estimated that the odds of developing RD increases by a factor of 1.147 with every passing year. The odds of RD were found to diminish by 0.653 with an increase in step of coloboma (according to Ida Mann classification) [51].

**Fig. 17:**
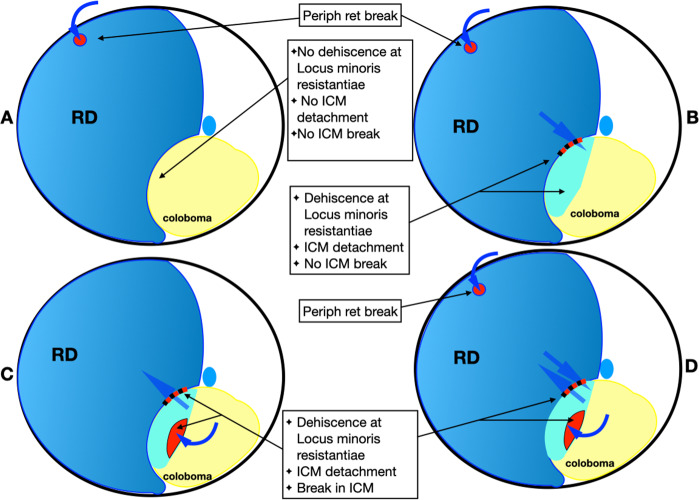
Illustrations demonstrating the mechanism of RD in eyes with coloboma. **A** Peripheral break present, ICM breaks absent and no dehiscence at Locus minoris resistantiae: only peripheral break contributes to the RD and RD does not extend into coloboma. **B** Peripheral break present, dehiscence at Locus minoris resistantiae present, ICM break absent: only peripheral break contributes to RD but RD extends into the coloboma. **C** ICM break present, dehiscence at Locus Minoris resistantiae present, peripheral retinal break absent: fluid spreads through ICM break to cause ICM detachment before spreading beyond coloboma to cause RD. **D** ICM break present, dehiscence at Locus minoris resistantiae present, peripheral retinal break present: fluid enters sub-retinal space both from peripheral retinal break and through break in ICM.

For the purpose of this discussion the term ‘RD’ is used to indicate separation of normal neuro-sensory retina (outside the coloboma) from the RPE. The term ‘ICM detachment’ is used to indicate elevation of the ICM from the coloboma floor. The following dehiscences in isolation or in combination need to be considered: (a) peripheral retinal breaks, (b) breaks in ICM and (c) dehiscence in the Locus minoris resistantiae. Figure 17 demonstrates pictorially the role played by each of the lesions and the combination of retinal and ICM detachment that occur consequently.

#### Isolated breaks in ICM

This situation can cause detachments of the ICM that do not spread beyond the coloboma border. These detachments remain asymptomatic and are not uncommonly seen in fellow eyes when patients come with symptoms in one eye. The risk of these becoming clinical RDs is significant and hence could be an important indication for prophylactic laser retinopexy.

#### Isolated breaks in peripheral retina

This situation is similar to RD in a non-colobomatous eye. The RD stops at border of the coloboma and there is no ICM detachment. Caveat: one must be certain that there is not even a rim of ICM detachment.

#### Isolated breaks in Locus minoris resistantiae

This situation is hypothetical and even if it can occur, would be of no clinical significance.

#### Breaks in peripheral retina and Locus minoris resistantiae

The RD will be associated with variable extent of ICM detachment and only a peripheral retinal break is identified.

#### Breaks in ICM and Locus minoris resistantiae

This is perhaps the commonest situation one comes across in coloboma-related RDs. When a communication between sub-ICM space and the sub-retinal space is established by way of breaks in the Locus minoris resistantiae, clinical RDs can occur. These are always accompanied by variable extent of ICM detachments.

#### Breaks in ICM, Locus minoris resistantiae and peripheral retinal breaks

The situation is similar to what was described above with the additional feature of presence of peripheral retinal breaks.

#### RD unrelated to coloboma

Faced with a RD in an eye with choroidal coloboma, the contribution of the coloboma to its causation needs to be determined in order to plan the appropriate management. Detachments of retina that do not cross the coloboma border are likely caused by a peripheral lesion. However, this judgement could be difficult clinically in some cases due to—(1) shallow ICM detachment restricted to periphery of coloboma. (2) Nystagmus. (3) Severely microphthalmic eyes that make it difficult to assess the fundus critically. When in doubt it is best to assume that coloboma is contributory to the causation of RD.

#### Acute vs. chronic vs. acute on chronic RD

RDs can be acute, chronic or acute on chronic. Often the area immediately beyond the coloboma has evidence of long-standing RD in the form of thin retina, RPE alterations, etc., and the detached retina beyond has the appearance of fresh RD. Chronic RDs are characterised by variable degree of PVR and thinning of the retina and sub-retinal gliosis.

#### Sub-clinical RD

A taut ICM can cause traction on the retina at the border of coloboma resulting in a shallow RD just beyond the border. This is not obvious on clinical examination but best detected on OCT (Figs. 11A, F) [23].

### Management of RD in eyes with coloboma of choroid

The management has evolved with the development of vitreo retinal surgical techniques. While scleral buckling was tried initially, it has been replaced by vitrectomy approach in most situations. A few reports have shown pneumatic retinopexy to be successful in select cases.

#### Pneumatic retinopexy

Isolated case reports refer to usage of this technique to reattach the retina followed by laser photocoagulation along the coloboma margin [52, 53]. It is obvious that this technique can only work in very selected cases and with a very compliant patient.

#### Scleral buckling

Before the advent of vitrectomy, scleral buckling was tried with predictably poor results. Jesberg and Schepens could achieve retinal reattachment in four of seven cases [50]. Understanding that the coloboma border holds the key to the causation of RDs, attempts were made to treat the border of coloboma with radial buckles [54, 55]. This orientation of two radially converging buckles was popularly called as Chinese figure of eight scleral buckles. In Wang’s series [55], six of the fourteen with figure of eight buckle had successful reattachment, while only one of the six eyes treated with conventional buckle could be reattached.

Understandably, scleral buckling is difficult. The posterior most part of the coloboma can never be adequately buckled. Attempting to close the ICM breaks is equally difficult. These breaks in the diaphanous tissue are difficult to identify and cryo has no effect in creating adhesion due to lack of choroid and RPE underneath. In addition, scleral buckling in a significantly microphthalmic eye is technically challenging.

However, peripheral scleral buckle can be the approach of choice for RD not attributable to coloboma, as long as one is confident of this assessment. This difficulty in assessment was brought out in a publication by Jalali et al. An initially planned peripheral scleral buckle was converted to vitrectomy, when ICM detachment and breaks could be identified—pointing to the role of coloboma in the causation of RD [56].

### Pars plana approach

The advent of vitrectomy has given us a direct approach to the area of interest. The magnification provided by the operating microscope facilitated the critical evaluation and identification of even a small strip of ICM detachment, and the identification of breaks in ICM. It facilitated the controlled treatment of the coloboma border with laser retinopexy and offered us long-term internal tamponade to facilitate the development of a firm adhesion along the border of coloboma. Gonvers first reported successful use of vitrectomy and silicone oil tamponade in a case of coloboma RD where prior buckling for a more obvious peripheral break failed [57].

### Surgical procedure

#### Role of additional encirclage

Placing an encircling band has no definite advantage in eyes with large colobomas that reach to the inferior periphery. In eyes with aphakia and pseudophakia, vitrectomy can be more thoroughly performed in the periphery and hence the role of encirclage is reduced further. However, in eyes with significant PVR, encirclage helps in the overall process of relief of traction.

#### Sclerotomies

Intuitively, one would place sclerotomies more anteriorly in microphthalmic eyes. However, it may be important to differentiate between microphthalmos and microcornea. In eyes with microcornea without microphthalmos, the limbus- ora distance is greater than normal [58].

#### Management of lens

In addition to eyes with cataract, a clear lens may sometimes need to be sacrificed in eyes with significant microphthalmos. In most cases one can remove the lens with the vitrector. However dense nuclear sclerosis is not an uncommon association of coloboma of choroid and may necessitate use of phaco fragmatome.

#### Vitrectomy

The posterior vitreous is not detached in most cases of coloboma-related RDs, although the vitreous is partly liquified in front of the coloboma. Inducing PVD could be tricky and routine injection of triamcinolone is recommended to aid this step. If simple suction with cutter fails to induce PVD, a pocket of vitreous detachment can be induced near the disc using vitreous forceps followed by use of suction. The peripheral vitreous should be debulked aided by scleral indentation.

#### Assessment of coloboma margin

Identification of the presence and extent of ICM detachment is possible more definitively under the magnification of the operating microscope. ICM breaks can also be clearly identified.

#### Approach in eyes with significant PVR

Membrane peeling techniques are similar to routine cases of PVR. If retinotomy is needed for sub-retinal band removal, considering the coloboma inferiorly and the limited functional retina available superiorly, these retinotomies should be placed as peripheral as possible.

#### Retinal reattachment

**Fig. 18:**
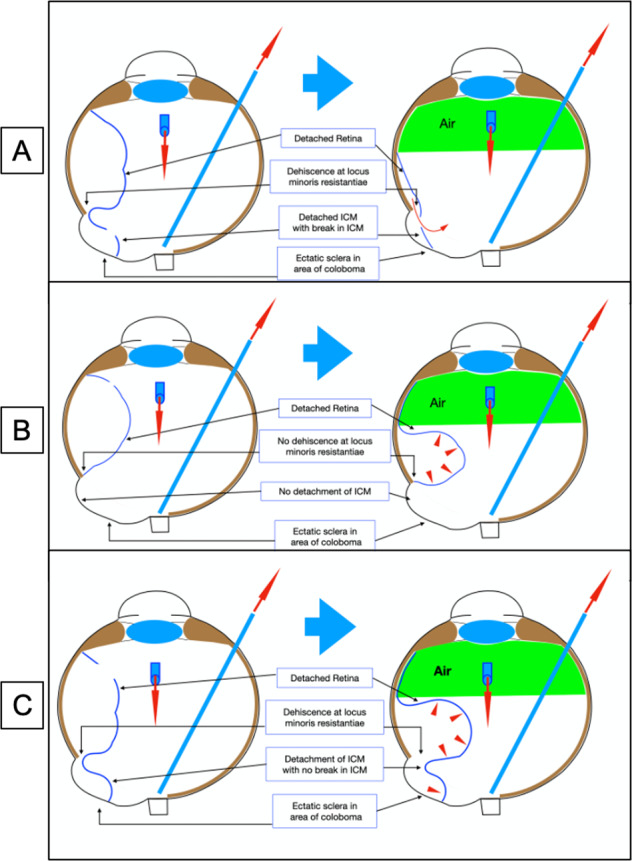
Illustrations demonstrating behaviour on fluid air exchange in colobomatous eyes with RD (with no attempt at sub-retinal fluid removal). **A** In the presence of ICM break and dehiscence at Locus minoris resistantiae, the sub-retinal fluid is pushed into the vitreous cavity by the air and retina flattens. **B** In the absence of dehiscence at Locus minoris resistantiae and absence of ICM break, the sub-retinal fluid collects around coloboma and balloons the retina. There is no ICM detachment. **C** In the presence of dehiscence at Locus minoris resistantiae but no ICM break, fluid collects around the coloboma and balloons the retina as well as ICM.

Simple fluid air exchange without any attempts at direct sub-retinal fluid removal, elegantly demonstrates the pathology in a given eye (Fig. 18).

#### Situation 1

ICM breaks + breaks in zone of least resistance ± peripheral breaks: on injection of air into vitreous cavity, the sub-retinal fluid is pushed into vitreous cavity through the ICM breaks. Thus, mere placement of the suction port over the coloboma area would result in flattening of the retina without need for drainage retinotomy (Fig. 18A).

#### Caveat

If the communication between the sub-retinal space and sub-ICM space is not large enough the fluid migrates slowly (especially if the sub-retinal fluid is thick) and hence can result in ballooning of the retina around the coloboma similar to situation 2 discussed below. However, one can differentiate between the two by observing the steady collection of fluid in the floor of coloboma even as it is being evacuated and the corresponding slow reduction in the bullous RD surrounding the coloboma.

#### Situation 2

If there is a peripheral break but no break at zone of least resistance, there will be no exit path for the sub-retinal fluid when air is injected. The fluid then collects around the coloboma margin resulting in ballooning of the retina. This does not flatten unless one sucks the sub-retinal fluid through a pre-existing peripheral break or a drainage retinotomy (Fig. 18B).

#### Situation 3

This is relatively rare. In the presence of a peripheral break and break in site of least resistance, but no ICM breaks, if one performs fluid air exchange, the retina and the connected ICM detachment would balloon but not flatten (Fig. 18C).

#### Retinopexy

In RDs not related to the coloboma, the retinopexy is strictly speaking needed only to the peripheral retinal breaks. In coloboma-related RDs, however, one has to treat the coloboma margin to be able to close the communications in the zone of least resistance. Theoretically, if one has the knowledge of the precise location of this communication, one can treat that segment of the coloboma margin alone. However, in most cases this information is not available. In addition, the induction of PVD can induce new tears in the zone of least resistance. Hence, it is advisable to treat the entire margin of coloboma. Even in eyes with RD not related to coloboma, if one is managing them through pars plana approach, it may be preferable to treat the coloboma margin as a measure of prophylaxis. Treatment of the ora serrata in the extra colobomatous area is optional. It serves to reduce risk of recurrences from unrecognized dialysis of ora serrata—especially in the meridian of the sclerotomy sites.

#### Difficulties in performing retinopexy along coloboma margin

(1) A taut ICM may make it difficult to get laser burns right at the coloboma margin, necessitating broader coverage.

(2) In eyes with fovea just outside the coloboma margin, broad treatment can potentially destroy the fovea. Hence one may have to spare the fovea and treat up to it on either side. The risk of recurrent RD exists when oil is removed if this untreated location harbours a communication between sub-retinal and sub-ICM space.

(3) Where the disc is involved in the coloboma, the functional border of the coloboma would need to be carefully treated with light burns to avoid nerve fibre layer damage. Use of diode laser is helpful to prevent accidental full thickness burns.

#### Internal tamponade

Long-term tamponade with silicone oil is desirable in most cases in view of (1) young age of the patients and consequent inability to enforce proper positioning, (2) the extent of area to be supported is fairly large and extends below the horizontal meridian on both sides. However, C_3_F_8_ tamponade may be acceptable in selected cases. When silicone oil is injected, 5000 centistokes may be preferred. Risk of emulsification is potentially more in these hyperactive children (mechanical agitation as a factor in oil emulsification) when low viscosity oil is used.

#### Role of relaxing incisions in the ICM

Combination of a taut ICM with breaks in ICM and a relatively deep ectasia of coloboma can result in the ICM getting lifted up like a trampoline. Fluid air exchange results in air entering the sub-ICM space even if the retina beyond the coloboma settles well. The concern would be whether this traction would not permit the laser-induced adhesion to take place along the coloboma margin. If felt necessary, this traction can be relieved by relaxing cuts on the ICM [59, 60]. However, one should be conscious of and avoid the major blood vessels that may traverse the ICM and supply the retina beyond.

### Results of management of coloboma-related RD

Hanneken reported successful outcomes in seven out of eight eyes who underwent pars plana vitrectomy [61]. Gopal et al. reported a series of 85 eyes of coloboma-related RDs that were managed with pars plana vitrectomy approach [62]. Silicone oil was used as internal tamponade in 94.1% eyes, while C_3_F_8_ gas was used in 5.9% eyes. Silicone oil removal was done in 80% eyes after an average duration of 4.7 months. The overall success rate was 81.17%. Re-detachment was seen in 16 eyes (3 out of 5 eyes with C3F8 and 13 out of 80 eyes with silicone oil tamponade). They reported raised intraocular pressure in 16% of eyes. Similar success rate (88.1%) was reported by Pal et al. in their series of 40 eyes with coloboma-related detachment [63]. Ten of the 21 eyes that underwent silicone oil removal in this series developed recurrent RD. Nagpal et al. in their series of 46 eyes reported a success rate of 88.9% [64]. In this series, 38 eyes underwent vitrectomy with silicone oil tamponade while 8 eyes had undergone scleral buckling procedure. A recent large multi-centric retrospective study of 119 eyes reported a final anatomical success of 87.4% [65]. In this study, 77.3% eyes had received silicone oil, 10.1% SF_6_ and 12.6% C_3_F_8_ as tamponade. SF_6_ use was associated with 50% risk of recurrence (6 out of 12 eyes).

It is evident that pars plana approach and silicone oil tamponade are probably needed in a majority of cases. Even with anatomical success rates of more than 80%, visual acuity better than 20/400 is reported only in 35.7–78.4% cases. The discordance between anatomical and functional success rate is attributable to the colobomatous involvement of the disc and macula.

### Post-silicone oil removal risk of recurrent RD

This is an important concern that makes surgeons procrastinate from removing silicone oil—resulting in increased risk of oil-induced complications. The important reasons for such recurrences are:

(1) Incomplete vitrectomy: failure to induce PVD can result in persistent unrecognized traction on the retina/coloboma margin that becomes manifest once oil is removed.

(2) Unrecognized dialysis of ora near sclerotomy sites.

(3) Unrecognized shallow RD beyond the coloboma margin: In the backdrop of lasered margin, a thin film of fluid may be missed. This could represent persistent communication between sub-ICM space and sub-retinal space resulting in recurrent RD once oil is removed. Pre-operative OCT when possible helps to avoid this pit fall. There could potentially be a role for intra-operative OCT.

The cause of this shallow detachment is often a taut ICM exerting traction on the retina. In this tug of war, a broader laser treatment (when possible) helps to reduce recurrent RD.

(4) Proliferative vitreo retinopathy.

(5) Casual approach during oil removal: one cannot overemphasize, the need to be diligent in evaluating the entire retina including the margin of the coloboma intra-operatively at time of oil removal. It gives valuable opportunity to correct the issue same time including reinjection of silicone oil when needed.

### Choroidal neovascularization (CNV)

Sub foveal CNV was reported as a complication of coloboma of choroid when the macula is close to the coloboma margin [66–69]. The CNV is usually classic and could be pigmented. Reported treatment approaches include laser photocoagulation, photodynamic therapy with Verteporfin, anti VEGF injections and surgical removal of the CNV.

### Scleral fistula

The thin ectatic sclera can give way spontaneously or after minor trauma leading to scleral fistula [70, 71]. These have been repaired with various techniques including fibrin glue, scleral patch graft, buckle etc. One case of spontaneous closure has also been reported [72]. These eyes present with low IOP and signs of hypotony maculopathy.

### Cataract

**Fig. 19:**
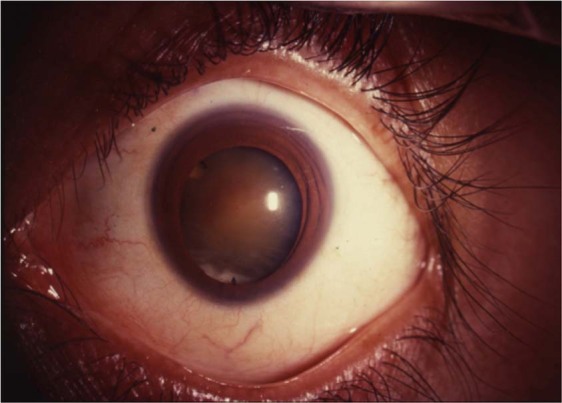
Slit-lamp photograph of an eye with partial coloboma iris. Note the dense nuclear cataract.

Uveal coloboma has been associated with early onset cataracts and typically significant nuclear cataract even in young age (Fig. 19). Mohamed et al. in a series of 145 eyes of 98 patients (mean age of 23 years) found cataract in 48.9% [73]. The commonest lens opacity noted was nuclear sclerosis in 51%. They observed a linear opacity in the region of coloboma in 29% of the cases and labelled it ‘coloboma cataract’. Dislocation or subluxation of the crystalline lens was found in a few eyes.

Reports indicate higher incidence of complications with cataract surgery in these eyes. Chaurasia et al. reported a series of 26 eyes that underwent cataract surgery [74]. They performed phaco emulsification for soft and moderately hard lenses but for very hard lenses opted for extra capsular cataract surgery. The intra-operative complications (8/26 eyes) included rhexis extension, posterior capsular rupture and vitreous loss. Phylactou et al. had a 19.5% incidence of posterior capsular tear with vitreous loss [75]. Khokhar et al. reported a series of 21 eyes that were successfully managed with phaco emulsification and IOL implantation aided by use of capsular and iris hooks [76]. They found the nuclei to be hard and leathery. They recommend orientation of the haptics perpendicular to the coloboma to get better centration. Kohli et al. reported a 9.3% incidence of complications after phaco emulsification or manual small incisional cataract surgery (M-SICS) in a series of 280 eyes of coloboma and cataract. They recommend phaco emulsification for softer cataracts and corneal diameter >8 mm. For smaller corneas and hard nuclear cataracts, M-SICS is recommended [77]. For extremely microphthalmic eyes, pars plana approach is recommended even for hard lenses in view of the small cornea and difficulty in performing surgery through limbus [78].

#### Repair of iris coloboma along with cataract surgery

There are reports of iris coloboma repair along with cataract surgery [79]. The avowed objective of this additional step is to reduce glare post-operatively. Khokhar et al. did not notice any complaints of glare or diplopia post-operatively although no attempt was made to repair the iris coloboma. One should be cognizant of the fact that these are patients born with the iris coloboma and, hence, are used to the glare (if any) from day 1 of their life. Hence repair of the iris coloboma if at all, has more of cosmetic than functional value.

### Prophylaxis against RD in eyes with coloboma of choroid

Considering the high incidence of RD in the lifetime of a patient with coloboma of the choroid, attempts at reducing the incidence of RD are important. There are, however, several issues with the prophylactic laser:

(1) Completeness of treatment: for the treatment to be effective, the entire coloboma margin must be surrounded by at least 2–3 rows of laser burns in an attempt to segregate the coloboma from the rest of the retina and not permit any breaks in the zone of least resistance and ICM to lead to clinical RD. This can safely be performed only in eyes with coloboma that do not involve the disc and macula and that have a considerable space between the coloboma margin and macula and disc. However, a majority of colobomatous eyes have the optic disc within the coloboma[19].

(2) Safety of laser treatment around the functional disc border: where the coloboma involves the optic disc, effective laser treatment would involve treating around the functional border of the optic disc. Inadvertently placed heavy burns in this area run the risk of nerve fibre layer damage. The ability to place precisely titrated burn intensity is low with indirect ophthalmoscopic delivery compared to slit-lamp delivery. Unfortunately, in children under anaesthesia, one can only use indirect ophthalmoscopic laser delivery.

Tripathi et al. reported their experience of prophylactic laser in 201 eyes with choroidal coloboma that were followed up for 3 years [80]. They describe four patterns of treatment. In three of them, some sectors of the coloboma margin were left untreated to protect the macula. The chances of this area harbouring the communication between sub-ICM space and sub-retinal space exists and this could negate the value of laser prophylaxis. In 3 years, they had two cases developing RD despite laser. In another series by Uhumwangho et al., the mean follow up was even shorter—1.59 years in treatment naïve patients vs. 0.79 years in laser-treated patient [51]. Thus, the efficacy of the laser treatment cannot be concluded from either of the studies in view of the rather short follow up, when one understands that risk of RD is cumulative with age.

A complete treatment around the coloboma, where possible should reduce the risk of RD considerably. However, there is no finite answer as to whether partial treatment of coloboma margin (as was done in the studies above) are of any value in reducing the risk of RD unless long-term studies are conducted.

## Conclusions

Ocular coloboma has a wide spectrum of presentation. While inherited cases and those with chromosomal abnormalities are well described, sporadic colobomata are more common where in environmental and maternal factors probably are responsible. Visual acuity could be affected by the coloboma itself if it involves disc and fovea or because of complications such as RD, choroidal neovascularisation, etc. Identification of ICM detachment is crucial to understand the contribution of coloboma in the causation of RD. Pars plana vitrectomy with silicone oil tamponade and endolaser along the coloboma margin is associated with good surgical success. In eyes with coloboma, cataract surgery is associated with greater than normal risk of complications.

Future research should address (a) better ways of identifying causes of non-syndromic coloboma. (b) Better imaging using swept source OCT with longer scans that can give panoramic understanding of the anatomy. (c) Randomized controlled trials to elucidate the prophylactic role of laser photocoagulation in preventing RD. (d) Randomized controlled trials to elucidate role of ideal internal tamponade in management of coloboma-related RDs.
